# A case of chronic total occlusion in popliteal artery recanalized by double snare piercing technique

**DOI:** 10.1186/s42155-023-00380-z

**Published:** 2023-06-22

**Authors:** Hirokazu Miyashita, Kazuki Tobita, Syuhei Uchida, Eiji Koyama, Yusuke Tamaki, Takayoshi Yamashita, Shigeru Saito

**Affiliations:** Department of Cardiology, Heart Center, Shonan Kamakura General Hospital, Okamoto 1370-1, 2478533 Kamakura, Japan

**Keywords:** Retrograde approach, Chronic total occlusion, Peripheral artery disease, Endovascular therapy

## Abstract

**Background:**

Although majority of cases with chronic total occlusion (CTO) in femoro-popliteal lesion were treated with antegrade approach only, some lesions require alternative approach due to its complexity. Bi-directional approach is useful on endovascular therapy (EVT) for CTO; however guidewire passage through the lesion is impossible in some challenging cases. The present case shows a successful re-entry technique utilizing two snare catheters from an antegrade and retrograde access site (double snare piecing technique).

**Case presentation:**

A 79-year-old woman with right leg intermittent claudication (Rutherford category IV), who had undergone unsuccessful EVT for popliteal CTO, required another EVT for the worsening symptom. Following the failed conventional crossing technique (wire knuckle technique, intravascular-ultrasound-guided wiring, and controlled antegrade and retrograde subintimal tracking technique), two snare catheters were placed and the snare loops were pierced by a puncture needle percutaneously. After an 0.014 wire was inserted into the needle, the needle was withdrawn. The wire was pulled from the retrograde side and was externalized. Then, the antegrade snare catheter was pulled and externalized, to make the wire across the lesion. After that, a microcatheter was advanced along the externalized wire from the retrograde side and cross the lesion. The wire was replaced with a new wire, which completely created pull-through system. After the hemostasis by balloon inflation and lesion preparation, this procedure was completed with an endoluminal-covered stent and two inter-woven stents. The re-entry site was covered by the inter-woven stent. Her symptoms improved after the procedure, and the lesion has not developed restenosis at 2-years follow-up.

**Conclusions:**

This re-entry technique of puncturing two snare loops (double snare piercing technique) might be effective for achieving successful passage through challenging femoropopliteal CTO cases.

**Supplementary Information:**

The online version contains supplementary material available at 10.1186/s42155-023-00380-z.

## Background

Endovascular therapy is recommended as the first-choice strategy for stenosis or occlusion shorter than 25 cm in lower extremity artery disease [1]. However, antegrade crossing techniques via femoral access could be challenging and may be associated with technical failure due to the complexity and calcifications of the lesion. Previous studies reported antegrade approach could result in failure in 10–20% of infra-inguinal chronic total occlusion (CTO) [2, 3]. Although the re-entry technique and devices developed, a few cases may result in failure to cross the occluded lesion.

We report here the case of a patient presenting complex popliteal CTO which was successfully treated with novel double snare piercing technique after conventional crossing technique failure.

## Case presentation

A 79-year-old woman without any atherosclerotic vascular risks complained right leg pain at rest (Rutherford category IV). She was admitted to our institution three years ago and underwent EVT two times (3 years ago, and 2 years ago), which were clinically and technically unsuccessful [4] due to poor below-the-knee arteries. After the EVTs, she has continued medical therapy. However, the symptoms have worsened, and she desire to undergo treatment again. She refused surgical treatment and decided to receive another EVT. Diagnostic ultrasonography showed total occlusion at distal SFA and the popliteal artery with popliteal artery dilatation, which suggested popliteal artery aneurysm. The end of the occlusion part was unclear.

**Fig. 1:**
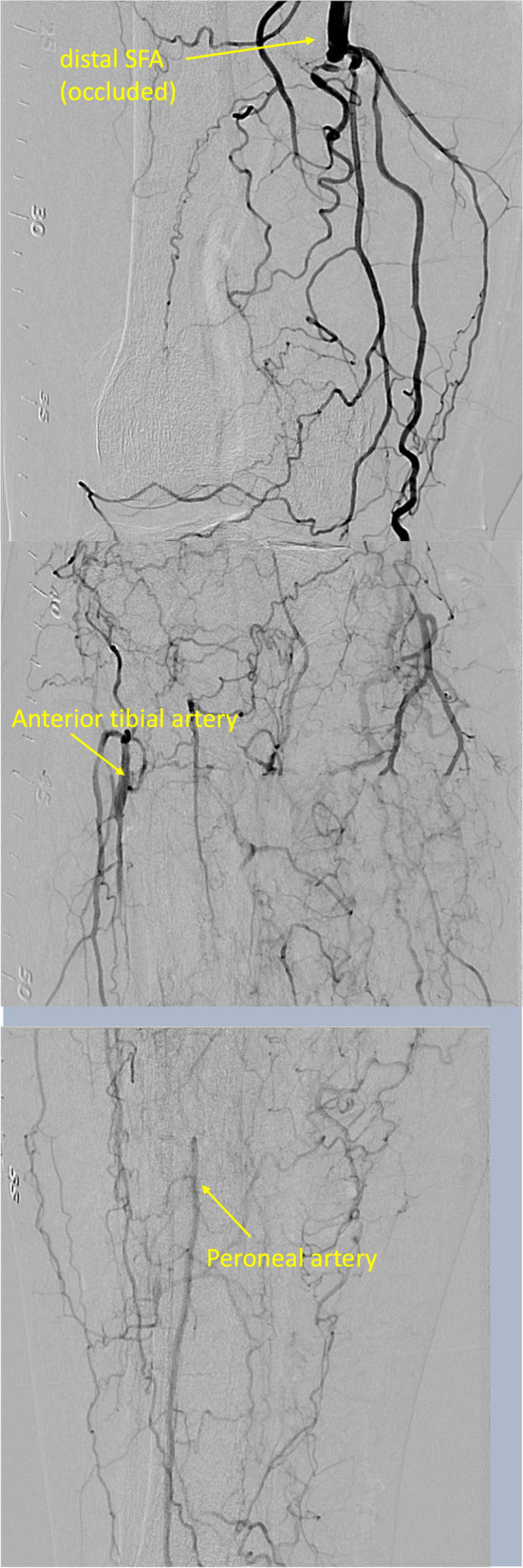
Initial angiography. Initial angiography demonstrated total occlusion from right distal superficial femoral artery to popliteal artery. Anterior tibial artery was enhanced via the collateral artery, while peroneal artery was not present at this level and enhanced at more distal level

**Fig. 2:**
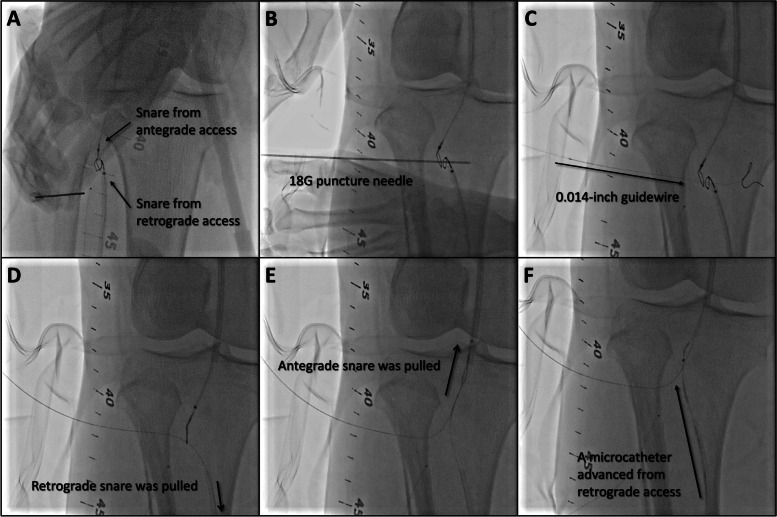
Double snare piercing technique. **A** Two snares from antegrade and retrograde access sites were placed in the same level and the two loops were aligned. **B** micro-puncture needle was inserted from the skin through the two loops. **C** A 0.014-inch guidewire was advanced after removing inner needle, and the outer needle was retrieved. **D **The retrograde snare grasped the wire and was pulled until the it was externalized, and the middle part of it came to the occluded lesion. **E** The antegrade snare was pulled so that the wire could cross the lesion. **F** A microcatheter was advanced along the externalized wire from retrograde side and crossed the lesion

**Fig. 3:**
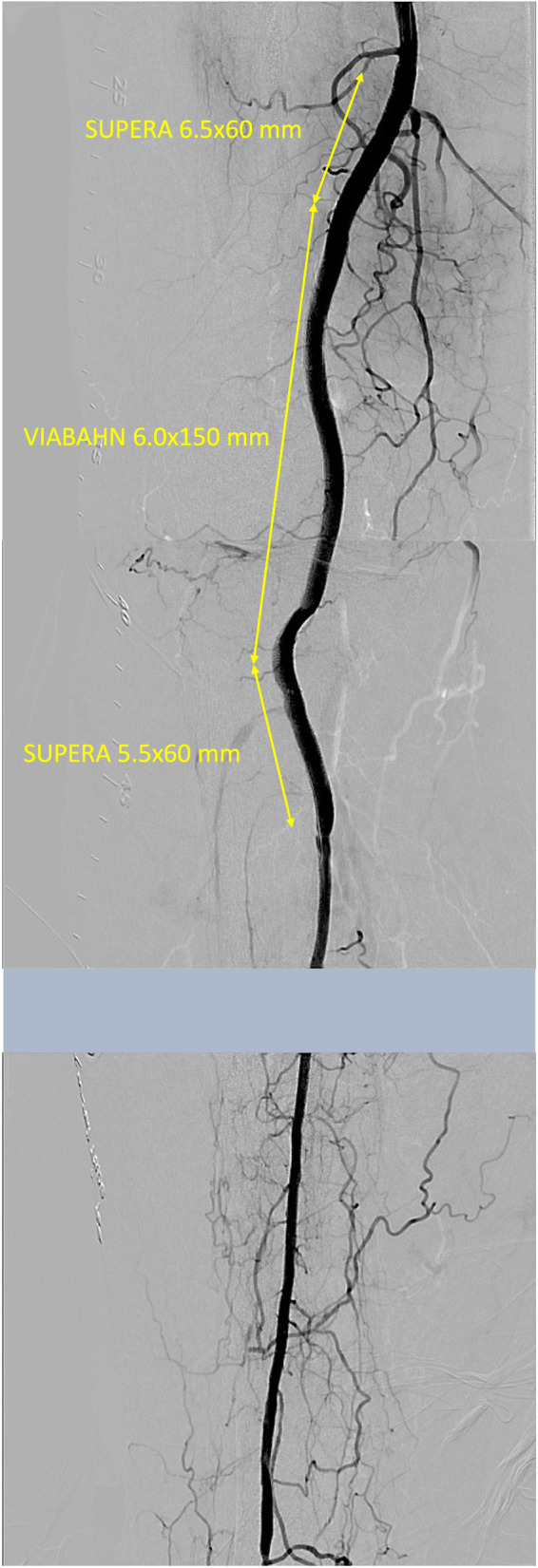
Final angiography. After an endoluminal bypass graft and two interwoven stents were implanted, angiography showed satisfactory result

An ipsilateral approach via femoral artery was established using 6 Fr SheathLess PV (ASAHI Intecc, Tokyo, Japan), and the distal peroneal artery, which was not seen in the second EVT, was observed as a puncturable site (Fig. 1). Therefore, the peroneal artery was punctured, and ICHIBANYARI (Kaneka Medix Corporation, Osaka, Japan) and Cruise (ASAHI Intecc co, Aichi, Japan) were inserted as the retrograde approach. Wire knuckle technique from both sides, intravascular-ultrasound-guided wiring from antegrade, and controlled antegrade and retrograde subintimal tracking were attempted. However, they failed because the occlusion site was too hard to cross. Another retrograde approach via proximal anterior tibial artery was done, which was also unsuccessful. Although the re-entry failed, antegrade and retrograde wires from peroneal artery were close to each other. Therefore, we inserted 5Fr sheath (Terumo, Tokyo, Japan), and 5 mm Goose Neck Snare (Medtronic, MN, US) catheters were placed from both sides. Then, both snares were aligned to be punctured percutaneously (Fig. 2A). An 18 G micro-puncture needle penetrated both snare loops percutaneously (Fig. 2B). After the inner needle was removed, 0.014-inch wire was inserted into the outer part (Fig. 2C). The retrograde snare grasped the wire and was pulled until it was externalized, and the middle part came to the occluded lesion (Fig. 2D). Then, the antegrade snare was pulled so that the wire could cross the lesion (Fig. 2E). SEEKER microcatheter (BD, NJ, USA) was advanced along the externalized wire and crossed the lesion (Fig. 2F). The wire was replaced by a new 0.014-inch Gladius MG14 PV ES (ASAHI Intecc co, Aichi, Japan) and the pull-through system was created. The schema of the technique is shown in Supplemental Fig. 1. (A) Two snare loops are placed in the same level. (B) A micro-puncture needle was inserted through the two loops percutaneously. (C) A 0.014-inch guide wire is advanced through the needle. (D) After removing the needle, the retrograde snare was pulled, while the antegrade snare was advanced to cross the occluded lesion. (E) The antegrade snare pulled the wire so that the wire crosses the occluded lesion. (F) A microcatheter was advanced along the externalized wire from retrograde side and crossed the lesion. A 4.0 mm SHIDEN HP (Kaneka Medix Corporation, Osaka, Japan) balloon was inflated for 10 min for lesion preparation and hemostasis. After the balloon dilatation, the hemostasis was achieved. The procedure was completed using an endoluminal stent-graft, VIABAHN (W. L. Gore & Associates, DE, US) 6.0 × 150 mm for popliteal artery, and two interwoven stents, SUPERA (Abbott, IL, US) 6.5 × 60 mm for SFA, and 5.5 × 60 mm for the tibio peroneal trunk, following pre-dilatation with a 6.0 mm non-compliant balloon. Final angiography revealed no complications (Fig. 3).

A duplex ultrasound on the day after the procedure showed patency of the superficial femoral artery and popliteal artery recanalization without any access-site complications. The symptoms improved after the EVT (Rutherford category I), and the patient did not develop restenosis at 2 years follow-up, which was confirmed by ultrasonography.

## Discussions

Previously, the snare piercing technique with a single antegrade snare was reported to be effective for femoropopliteal CTO cases with difficulty re-entering from subintimal space to true distal lumen [5]. Our technique modified this technique; we placed two snares antegradely and retrogradely and positioned the two loops on the same level. Thus, the snare piercing technique could be performed whenever the subintimal approach from both sides gets close regardless of the presence of lesion calcification. In addition, the double snare piecing technique can be effective when the antegrade wiring cannot reach the true distal lumen level; however, retrograde sheath insertion is required, which could be invasive. On the other hand, the single snare piercing technique require the antegrade wiring to that level. In our institution, the antegrade approach and conventional retrograde approach were attempted as the first strategy for CTO lesions. When these approaches were unsuccessful, the double snare piercing technique or single snare piercing technique were performed. Although the decision was at discretion of the attending physician, our institution had 4 cases of double snare piercing technique, all of which were technically and clinically successful and uneventful. A larger series and longer follow-up are warranted to establish the safety and efficacy of this technique.

## Conclusion

The current case demonstrated a successful treatment for popliteal artery CTO with double snare piercing technique, which might be one option for challenging femoropopliteal CTO cases.

## Supplementary Information

**Additional file 1: Supplemental figure 1.** Schema of double snare piercing technique. A. Two snares were placed in the same level. B. A micro-puncture needle was inserted through the two loops. C. A 0.014-inch guidewire was advanced. D. After removing the needle, the retrograde snare was pulled, while the antegrade snare was advanced to cross the occluded lesion. E. The antegrade snare pulled the wire so that the wire crosses the occluded lesion. F. A microcatheter was advanced along the externalized wire from retrograde side and crossed the lesion.

## Data Availability

The data and material on the case report is available from the corresponding author, KT, upon reasonable request.

## Abbreviations

CTO: Chronic total occlusion
EVT: Endovascular therapy
