# Supplementary figures and images for: A case of chronic total occlusion in popliteal artery recanalized by double snare piercing technique
Source: CVIR Endovasc. 2023 Jun 22;6:34. doi: 10.1186/s42155-023-00380-z (PMC10287857; doi:10.1186/s42155-023-00380-z)

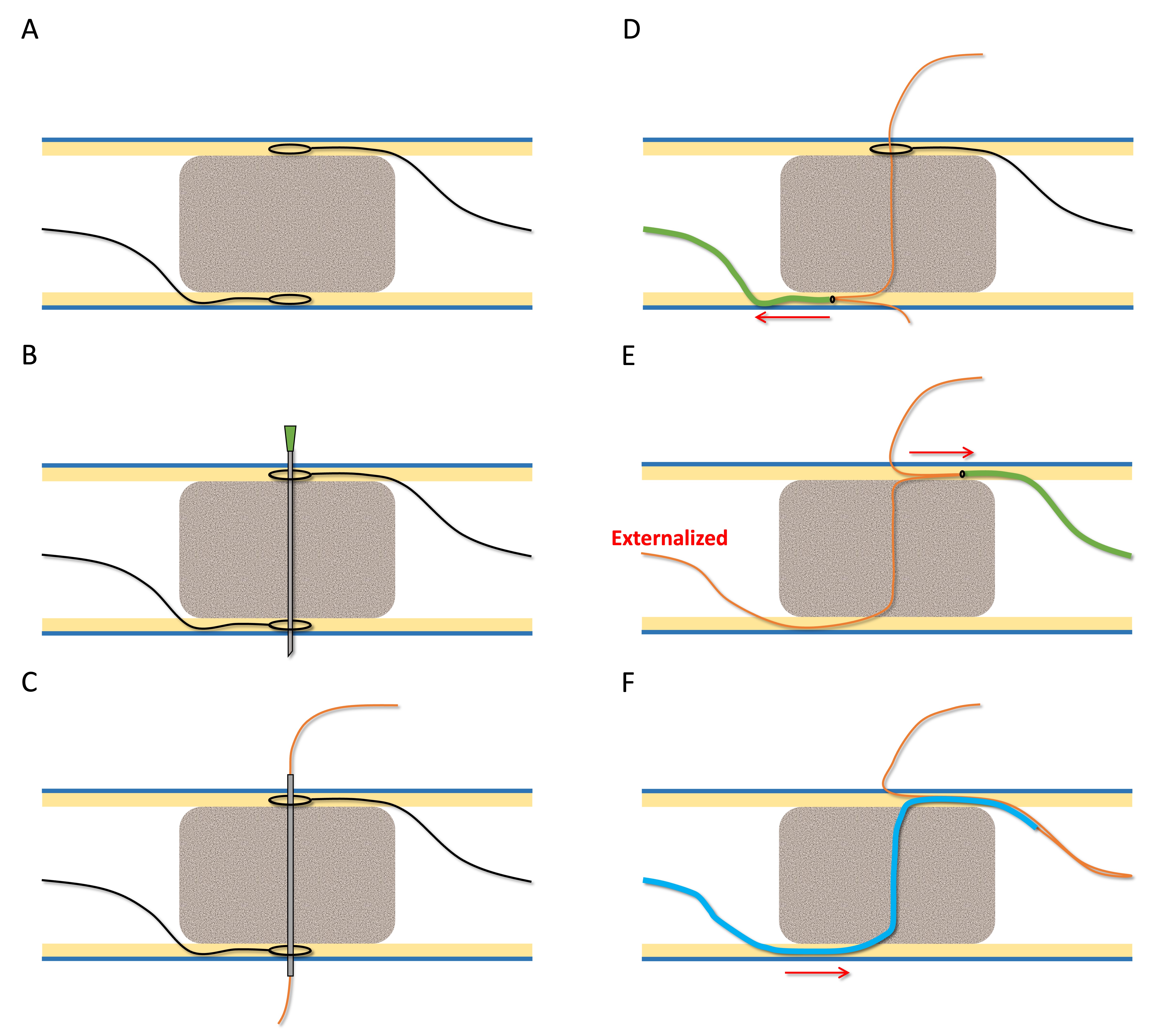

Supplement: Supplementary file 1 — Additional file 1: Supplemental figure 1. Schema of double snare piercing technique. A. Two snares were placed in the same level. B. A micro-puncture needle was inserted through the two loops. C. A 0.014-inch guidewire was advanced. D. After removing the needle, the retrograde snare was pulled, while the antegrade snare was advanced to cross the occluded lesion. E. The antegrade snare pulled the wire so that the wire crosses the occluded lesion. F. A microcatheter was advanced along the externalized wire from retrograde side and crossed the lesion. [file 42155_2023_380_MOESM1_ESM.jpg]
